# Unpacking the Black Box of Implementation: The Next Generation for Policy, Research and Practice

**DOI:** 10.1007/s10488-013-0512-6

**Published:** 2013-08-14

**Authors:** Kimberly Hoagwood, Marc Atkins, Nicholas Ialongo

**Affiliations:** 1 Department of Child and Adolescent Psychiatry, New York University Child Study Center, New York University School of Medicine, One Park Avenue at East 33rd, 8th Floor, New York, NY 10016 USA; 2 University of Illinois at Chicago, Chicago, IL USA; 3 John’s Hopkins Bloomberg School of Public Health, Baltimore, MD USA

It was, as best as we can recall, a late spring afternoon and the three of us were completing another of our semi-regular phone calls. As each of us is directing NIMH-funded Services Research Centers related to children’s mental health research, we often sought each other’s advice and feedback on the direction of our work. We saw the complementarity of our work: We all focused on low income and largely inner city populations; we all focused on schools and community agencies; we all focused on improving effective services via training, consultation, and fitting effective practices into natural community ecologies. We all brought different strengths: Nick’s work on specific evidence-based preventive services and their installation with fidelity; Marc’s work on building onto existing naturalistic supports in schools and agencies, rather than superimposing packaged practices; and Kimberly’s work on studying feasible and practical implementation strategies that can be adopted widely by states and healthcare systems.

On one of our calls among the three of us, we were discussing the need for synergy among our Centers and after bantering around several ideas, one of us called the question: Great ideas but who is going to really do this work? It was clear that the three of us in our roles directing these Centers had core research tasks to accomplish and little room for new projects. Yet, we saw there was an opportunity for pushing the research agenda beyond each of our Center’s mandates. Using the gestalt of our combined work to launch new projects. What if, one of us suggested, we brought our colleagues from each of our Centers together? And what if we focused not on our senior colleagues, who though brilliant were similarly preoccupied with their own core tasks? What if we brought together the extraordinarily talented early career faculty, postdocs, and graduate students from each of our Centers?

We agreed to host a series of cross-center meetings. The first was convened in Chicago in June 2011; the second in New York September 2011; the third in Baltimore October 2011; and the fourth in New York December 2012. The format was simple: the hosting center would present the key questions and data from its projects and the rest of the time would be spent brainstorming and networking our early career colleagues. Over time the long sought synergies became apparent; drafts of grants and papers were exchanged and ideas for new projects emerged. By the time of the second meeting, the group had formed working groups and from this emerged enough data based papers for special issues to develop including the one in this volume.

As we review these papers, we reflect back to our initial discussions and think about the new directions these papers represent. We want to note especially what we see as implications for policy, new research directions, and practice.

As has been said before, the ethical and scientific challenge for our field inheres in the sluggish movement of effective practices for children and families into the systems that are tasked to serve them (see Bickman and Hoagwood 2010). And, as has been documented many times, knowledge about how to improve the lives of children is available now and increasingly so in the form of packaged programs, and widely available trainings, aps and the like. The problem is not lack of what to do; it is lack of a systematic and evidence-informed way of doing it. And an overriding obstacle has been the intractable fragmentation of the service systems.

Consider a recent meta-analysis of treatment effectiveness studies for children’s mental health problems (Lee et al. 2013). They conducted a comprehensive literature search and identified 20 studies since 2007 that examined the effectiveness of interventions for anxiety, depression, and disruptive behavior problems conducted in practice settings. They then compared results from these effectiveness studies to benchmarks from two meta-analyses of efficacy trials. They found that for internalizing problems improvement rates for the effectiveness studies *matched* the two efficacy benchmarks. For disruptive behavior, results were more variable but were generally favorable with most studies *out*-*performing* the benchmark while a few under-performed. The authors note: “It is particularly noteworthy that the majority of the studies we reviewed addressed the transportability of interventions developed in North America to other countries, and for many, it included translation of materials into another language. *Only two of the studies were based in North America”* (Lee et al. 2013, p. 85, italics added). Thus, we, in the U.S. have developed a research enterprise that has led to impressive improvements in the treatment of children’s mental health problems *everywhere but here*!

Why is that? Undoubtedly there are many reasons but one is likely to be the excessive complexity of the child service systems to which these interventions are transported. Consider the implementation challenges that are increasingly documented in studies that examine the installation of evidence-based practices; they include regulatory constraints, lack of workforce training, inadequate supervisory structures, inability to bill for new practices, etc. (Bickman and Hoagwood 2010; Hoagwood et al. 2013; McHugh and Barlow 2010).

However, the traditional model for the mental health service system is uniquely poised for change. The political and social will exists now to make system changes on a massive scale. These changes have the potential to create a more integrated and data-driven health and mental health system. The 2010 Patient Protection and Affordable Care Act are creating a set of incentives, payment mechanisms, and attention to quality metrics that are restructuring the healthcare systems by which services are delivered. Because the umbrella of the healthcare act includes mental health and addiction services, along with a broad range of other health services, the *potential* for integrated services informed by data about outcomes and quality that are shared exists for the first time in this country (Berenson et al. 2013; Conway et al. 2013; Koh and Sebelius 2010).

Measuring and tracking quality indicators, for example, has been endorsed in the National Quality Strategy (NQS) of the Affordable Care Act, and developing child health care quality measures for use in Medicaid and the Children’s Health Insurance Programs (CHIP) has been mandated by the Children’s Health Insurance Program Reauthorization Act of 2009 (CHIPRA). Significant funding to support these initiatives exists. The Pediatric Quality Measures Program was allocated 40 million dollars to support seven Centers of Excellence in 2010 and to develop new measures and refine existing ones for a core set. The Centers for Medicare & Medicaid Services (CMS) also funded 10 five-year demonstration projects at an estimated total cost of $100 million in 2010, of which seven propose to develop, test, evaluate and/or report adherence to quality measures. The use of these quality indicators or measures will be sustained via financial incentives to collect and report on adherence rates through a federal match that is part of the American Recovery and Reinvestment Act of 2009 (ARRA). Eligible providers will receive these payments for demonstrating “meaningful use” of quality measures under the Electronic Health Records Incentive Program and will be able to benchmark their own performance against aggregated data (Conway et al. 2013; Zima et al. 2013). The point is that these massive healthcare policy changes are driving system-level changes. If we in the children’s mental health field use this opportunity to craft our research to inform these changes, then the possibility exists for a quality-driven, evidence-based national system of child health care.

The papers in this special issue focus on some of the important ingredients of quality change in children’s services, i.e., data-based strategies to improve the use of evidence-based prevention and clinical practices by teachers, counselors, families, and therapists after training. All of the papers focus on consultation, coaching, and other post-training strategies that can be delivered within the naturalistic settings of schools and community agencies. The papers pull apart the active ingredients that will yield higher fidelity to the effective practices and will improve outcomes. They also illustrate the complexity of the social and organizational processes that need attention if installation of effective practices is to be sustained.

For example, Becker et al. (2013) provide data from a large prevention trial that examines coaching visits to teachers who had been taught the Good Behavior Game to identify specific coaching strategies, and found that coaches strategically varied their use of strategies (e.g., modeling, delivery) based on teacher implementation quality. Coaching was associated with improved implementation quality. Similarly, Reinke et al. (2013) describe two understudied facets of fidelity: ratings of teacher engagement and differentiation of exposure to coaching. They show how these can be operationalized and measured so that they can be included in professional development of teachers and potentially used to establish benchmarks or standards for evaluating fidelity in evidence-based interventions.

Beidas et al. (2013) describes three mechanisms through which consultation may affect adherence and skill: connectedness, authenticity, and responsiveness. The analysis, using mixed methods, also suggests that active learning is not consistently the mechanism through which effective consultation operates. This leads to an important set of questions for further research.

Bearman et al. (2013) identify and test several specific predictors of evidence-based practice (EBP) use through a study of the components of effective supervision. They found that supervision involving modeling and role playing predicted higher EB practice use than discussion but also found age and sex-related differences. Because there is some evidence that didactic trainings without behavioral rehearsal or ongoing support are not sufficient to change therapist behavior, this study is important in suggesting that modeling and role-play may be two important behaviors to include in training and supervision of EBPs, and that therapists in community practice are able to implement these practices.

The other papers in the series also identify active ingredients of installing EBPs via consultation. For example, Edmunds et al. (2013) describe how behavioral rehearsal as a form of active learning may affect use of therapist skills. The degree of participation in the consultation process moderated the relationship between behavioral rehearsal and skill. In another study using qualitative data, Lyon et al. (2013) examines agreement to participate in training/consultation in EBPs in schools and identified a set of motivational factors based on social learning theory that were relevant. These included expectations, attitudes, as well as practical issues, such as time.

Masia-Warner et al. (2013) describe how specific consultation strategies can support school counselor’s implementation of an EBT for adolescent social anxiety by school counselors. They developed measures of adherence and competence and showed that agreement between counselors and consultants was strong for adherence but less strong for competence. Interestingly, regarding competence, counselors were observed by consultants to be good implementers of exposure exercises but less strong implementers of cognitive elements of the intervention. This provides a strong rationale for a multi-tiered intervention in which counselors work collaboratively with other mental health staff trained in more complex intervention strategies for those youth who require more intensive interventions.

Finally, Nadeem et al. (2013) describe the distribution of content and time in real-world supervision of therapists trained in EBTs. Importantly about one-fourth of the time is spent on administrative and organizational barriers, and 50 % on clinical content.

The implications of these papers for the new world order of healthcare policy reform are three fold. First, they demonstrate that identification, measurement, and testing of specific consultation practices after EBP training are feasible in real-world settings such as schools and community agencies. This is critical for future benchmarking of service quality. Much more work is needed, but these are important first steps. Second, they demonstrate the range of relevant consultation strategies and techniques that require further study to better improve not just the processes of EBP service installation but more importantly the outcomes. Third, they show how a new generation of research and of exceptionally promising junior researchers can help to mold the field of children’s services to make its yield directly applicable to important mental health policy issues.

These papers also point to new directions for research and practice. In regard to research design, as Proctor and Rosen (2008) note, service system research should involve the perspective of clinicians who make ideographic decisions regarding research evidence. Thus, an important next step in implementation and dissemination research is to match research designs with the intended use of the data to inform practice. Toward that end, innovative research designs that are both contextually relevant and methodologically rigorous are necessary to promote a clearer understanding of contextual factors that impede or enhance implementation processes. The advantage of these design alternatives is that the false dichotomy of ivory tower priorities for certainty and practice setting priorities for relevance is replaced by designs that accommodate the goal of advancing evidence-informed practice.

For example, Glasgow et al. (2005) recommend expanding the CONSORT criteria (that focuses primarily on enhancing internal validity) to include external validity criteria for “practical clinical designs.” They discuss the importance of representative sampling (including setting-specific factors), use of clinically relevant alternative interventions in place of no-treatment controls, and use of a broad range of relevant outcomes. Interestingly, included among the recommendations is the use of single subject designs, which have all but disappeared from clinical research. Recently, Kratochwill and Levin (2010) described procedures to adapt single-subject designs to accommodate randomized controlled trials. Specifically, they presented a model involving four stages of educational interventions with the goal to inform classroom practice that has relevance for mental health practice as well (see Fig. 4, p. 131). This is followed by a series of randomization strategies across units, settings, behaviors, or phases of intervention. Taken together, these strategies have the advantage of enhancing scientific rigor without sacrificing relevance to practice settings.

Another design issue that is highly relevant to dissemination and implementation research is the need for alternatives to the randomized controlled design when random assignment is not feasible. West et al. (2008) describe models that approximate random assignment for these occasions. Two categories are described, randomized encouragement designs that incorporate participant choice into the design (see also Freedman 1987 and Lavori et al. 2001 for a discussion of clinical equipoise) and quantitative assignment designs in which participants are assigned by preconceived criteria (e.g., risk or need). Finally, mixed method or hybrid research designs are also highly relevant to dissemination and implementation research. These designs can be important to allow an iterative process of research and practice can include both formative and generative research designs (Atkins et al. 2006). Other examples of hybrid designs include studies that incorporate aspects of effectiveness and implementation—to simultaneously test the impact of interventions under real world conditions (effectiveness) and test the spread or disseminability of these interventions (implementation) (see Curran et al. 2012).

The papers in this special issue also raise higher order questions about practice improvement. This has been an under researched area, and these papers fill a large hole. The issues raised are both micro and macro-level. Beginning at the micro-level, do some skills prove more trainable than others? For example, while training teachers (see Becker et al. 2013) in an urban school district to praise student behavior has proven to be no easy task, an even more difficult task has been training them to deliver the praise in a sincere and enthusiastic manner. Can sincerity and enthusiasm be trained? Are these personality traits that teachers or clinicians bring to the proverbial “table” and no amount of behavioral rehearsal will alter? Can these traits and/or aptitudes be reliably assessed and used in selecting candidates for training as clinicians and teachers? These questions lead to a natural set of research questions for future studies.

Moving to the more macro-level, several of these papers suggest that training and mentoring practices may need to be tailored to reflect variation in learning styles and clinician characteristics, such as gender and age. The influence of school, agency, or organizational context on training and consultation is also a practice question with researchable potential. Much has been written about the impact of social organizational context on uptake of new practices, on job satisfaction, and on child outcomes (see Glisson et al. 2012; Glisson and Schoenwald 2005; Glisson et al. 2010; Glisson et al. 2008). To make new practices stick in real world contexts, the combined influence of learning styles, clinician characteristics, and characteristics of the workplace need to be disaggregated. Core components that are modifiable need to be identified for the development of practice-based and targeted interventions.

While moving clinical science training programs towards the use of evidence-based training and mentoring practices is a formidable task, re-tooling via training and mentoring community-based clinicians in such practices is expensive, labor-intensive, and ultimately inefficient. An important question for improving practice relates to the kinds of institutional supports that will be needed to support these improvements. To this end, the common elements approach of Chorpita and Daleiden (2009) reflects some of the most original thinking about practical ways to advance practice improvement in children’s mental health. It is likely that in the re-tooling of the workforce web-based technologies are likely to provide valuable solutions. This will include web-based training and consultation models; the use of data to drive decision-making; the development of practical and robust metrics and measures that are sensitive to change, individually focused, and measurable (Bickman et al. 2012; Chorpita and Daleiden 2009).

The issue of embedding these web-based tools into real world practice settings raises its own set of implementation challenges (Bickman et al. 2012) and yet another research agenda. But it is important that the development and testing of these practical tools be done by people knowledgeable about mental health systems, fidelity to evidence-based practices, and meaningful child and family outcomes. If we don’t do it, someone else will.

In summary, the papers in this special issue advance the field of implementation science in children’s mental health by addressing real-world, practical, and down-to-earth issues about how best to train, coach, mentor, and provide consultation to front-line providers (teachers, counselors, clinicians, case workers) on alternative practices that are likely to improve child and family outcomes. The editors and all of the authors are to be commended for looking at the horizon and flying towards it with vision and hard work. The papers as a whole provide a picture of the future. Together they set a standard for linking policy, research and practice as they relate to evidence-based training and consultation methods to improve children’s mental health outcomes.
